# LECT2, A Novel and Direct Biomarker of Liver Fibrosis in Patients With CHB

**DOI:** 10.3389/fmolb.2021.749648

**Published:** 2021-09-22

**Authors:** Honghai Xu, Xutong Li, Zihao Wu, Linyan Zhao, Jiapei Shen, Jiaying Liu, Jiangfeng Qin, Yuanlong Shen, Jing Ke, Yuanyuan Wei, Jiabin Li, Yufeng Gao

**Affiliations:** ^1^ Department of Pathology, The First Affiliated Hospital of Anhui Medical University, Hefei, China; ^2^ Department of Infectious Diseases, The First Affiliated Hospital of Anhui Medical University, Hefei, China; ^3^ Department of Pathology, The Forth Affiliated Hospital of Anhui Medical University, Hefei, China; ^4^ Department of Hospital Infection Prevention and Control, The First Affiliated Hospital of Anhui Medical University, Hefei, China

**Keywords:** LECT2, direct biomarker, diagnosis, liver fibrosis, CHB

## Abstract

Chronic hepatitis B (CHB) patients with severe liver fibrosis would be more likely to progress to a poorer prognosis. Treatment is considered once the liver fibrosis reaches significant liver fibrosis (≥S2). Leukocyte cell-derived chemotaxin-2 (LECT2) has been shown to contribute to liver fibrosis progression. No research has focused on the role of LECT2 in liver fibrosis in CHB patients. This study enrolled 227 CHB patients and divided them into the training group (*n* = 147) and validation group (*n* = 80), respectively. The expression of LECT2 in serum, protein and mRNA of the human liver tissues was detected to analyze the possible associations between LECT2 and liver fibrosis. A receiver operating characteristic curve (ROC) was used to estimate the efficacy of LECT2 for predicting liver fibrosis. The data showed that there was a positive relationship between LECT2 and the progression of liver fibrosis. In the training group, LECT2 was demonstrated to have better effectiveness than APRI and FIB-4. The AUC was 0.861, 0.698, and 0.734 for significant liver fibrosis, and 0.855, 0.769, and 0.752 for advanced liver fibrosis. Besides, the efficacy of LECT2 in different statuses of patients with CHB was examined and the effectiveness of LECT2 had also been confirmed in the validation group. All the results confirmed that LECT2 could act as a perfect predictor and thus offers a novel and direct biomarker to estimate liver fibrosis more accurately.

## Introduction

Patients with chronic hepatitis B (CHB) represent an escalating worldwide health concern (Polaris Observatory Collaborators, 2018; Moon et al., 2020). The World Health Organization (WHO) reported that during 2019, there were 296 million people with CHB and 1.5 million people are newly diagnosed with CHB worldwide (WHO, 2021). A total of 15–40% of CHB patients may develop complications, such as liver fibrosis, liver cirrhosis, and liver cancer (WHO, 2017; Ma et al., 2021). Every year, an estimated 1.34 million people die from CHB or chronic hepatitis C, so the WHO called for achieving a realistic goal: “In 2030, the hepatitis-related mortality and new hepatitis-infections could be reduced 65 and 90%” (WHO, 2016; Cooke et al., 2019). The worse prognosis of patients with CHB has been related to an aggravated liver fibrosis progression (Dulai et al., 2017), and the guidelines of CHB had mentioned that it is an indication for antiviral therapy once patients have been diagnosed with moderate and above fibrosis (Terrault et al., 2018). Considering the significant impact of liver fibrosis, it is very necessary for the progression of liver fibrosis to be accurately described as soon as possible (Revill et al., 2019; Berumen et al., 2021).

Liver histology is the classic diagnostic method to estimate the progression of liver fibrosis which relied on professional pathologists, because it is time-consuming, invasive, difficult to operate, and expensive, and poses a variety of complications, the application of liver biopsy is limited in clinic (McGill et al., 1990). Several noninvasive methods have been reported that could be used in evaluating the severity of liver fibrosis (Cao et al., 2020; Loomba and Adams, 2020). One of the most common noninvasive scoring systems is the aspartate aminotransferase-to-platelet ratio index (APRI) and the other is fibrosis index based on the four factors (FIB-4), both of them have been indicated to predict severe liver fibrosis or liver cirrhosis (Wai et al., 2003; Vallet-Pichard et al., 2007). However, these tests relied on some indirect markers of liver fibrosis, including liver biochemical profile and clinical parameters (Bertrais et al., 2017). This makes it impossible for APRI and FIB-4 to accurately evaluate the degree of liver fibrosis once these indirect makers are too high or too low. Some studies have reported the low efficacy of APRI and FIB-4 and confirmed this statement (Jia et al., 2015; Singh et al., 2017).

It has been reported that Leukocyte cell-derived chemotaxin 2 (LECT2) is involved in immune reactions (Jung et al., 2018; Lu et al., 2020), severe liver injury (Segawa et al., 2001; Okumura et al., 2017; Slowik et al., 2019), cancer (L'Hermitte et al., 2019), nonalcoholic steatohepatitis (NASH) (Takata et al., 2021), nonalcoholic fatty liver disease (NAFLD) (Yoo et al., 2017) and so on. Our previous study reported the phenomenon that LECT2 played an important role in promoting the aggravation of liver fibrosis, and liver cirrhosis patients showed more concentration of LECT2 in the serum (Xu et al., 2019). However, no one had analyzed the association between LECT2 and liver fibrosis in CHB patients. Consequently, our group aimed to detect whether the expression of LECT2 was associated with liver fibrosis in CHB patients and confirm the effectiveness of LCET2 for predicting liver fibrosis.

## Methods and Materials

### Patients and Criteria

From August 2018 to August 2021, 227 patients with CHB passed the review of the inclusion and exclusion criteria and were recruited in this study. The training group enrolled 147 patients and the validation group enrolled 80 patients. The inclusion criteria were: 1) the diagnosis of CHB is consistent with the American Association for the Study of Liver Diseases (AASLD) 2018 hepatitis B guidance (Terrault et al., 2018); 2) patients were treatment-naive; 3) patients had undergone liver biopsy in the Department of Infectious Diseases, the First Affiliated Hospital of Anhui Medical University. The exclusion criteria were: 1) underlying liver diseases, such as chronic hepatitis C, chronic hepatitis D, autoimmune liver disease; 2) alcohol consumption, alcohol-related diseases; 3) obesity, NASH, NAFLD; 4) complications of systemic diseases, such as digestive system disease, cardiovascular system disease, and rheumatic immune system disease. This study was performed following the Declaration of Helsinki and was approved by the Ethical Committee of the First Affiliated Hospital of Anhui Medical University, Hefei, China.

### Laboratory Tests and Noninvasive Scoring Systems

Blood samples were collected and performed on the day of the liver biopsy. Two general validated noninvasive scoring systems were calculated using the original equations (Wai et al., 2003; Sterling et al., 2006). In the calculation of APRI, the upper limit of normal (ULN) for AST level was defined as 50 IU/L.

$$APRI=\frac{\frac{AST}{ULN}\;}{platelets}*100$$

FIB−4=age years∗AST/(platelets∗(ALT^(1/2)))

### Test for LECT2

The serum level of LECT2 in patients with CHB was measured by ELISA, as previously reported (Xu et al., 2019). The assay kits were purchased from Wuhan USCN Business (Cat No: SEF541Hu). RNA *in situ* hybridization (ISH) was completed using an RNAscope^®^ 2.5 HD Duplex Assay manual kit (ACDBio). RNA probes for LECT2 were customer-designed at ACDBio (Newark, CA, United States). LECT2 protein levels in various stages of liver fibrosis were detected by immunohistochemical (IHC) staining. A LECT2 monoclonal antibody (Cat No: Sc-398071) was purchased from Santa Cruz Biotechnology.

### Liver Histology

Liver tissues were obtained by liver biopsy using ultrasound-guided needles in patients with CHB. The progression of liver fibrosis was assessed by two experienced pathologists through the Scheuer scoring system, the description from S0 to S4 represents the aggravation of liver fibrosis (Yan et al., 2020). The patients were classified as having significant liver fibrosis when the fibrosis stage was ≥S2 and advanced liver fibrosis when the stage was ≥S3.

### Statistical Analysis

Spss 22.0 statistical software package (SPSS Inc., Chicago, Illinois, United States) was adopted for data analysis. The categorial parameters and continuous parameters were expressed as numbers or median and quartiles. Chi-square test and Fisher’s exact test were used to analyze the difference in the number of different liver fibrosis stages and patients with HBeAg-negative between the training group and validation group, and the Mann-Whitney U-test was used to determine intergroup differences of continuous data. Kruskal-Wallis H-test was used to compare the expression of these predictors in various stages of liver fibrosis. Spearman correlation coefficient was used to count the relationship between liver fibrosis and the predictors. The univariate and multivariate regression analyses were used to analyze independent influencing factors. The diagnostic efficacy of LECT2, APRI, and FIB-4 was evaluated by the receiver operating characteristic curve (ROC) and the area under the ROC (AUC). The AUC of LECT2, APRI, and FIB-4 were compared using the method of DeLong et al. (Demler et al., 2012). A two-sided *p* value of <0.05 was deemed statistically significant.

## Results

### Patient Clinical Characteristics

The demographic and laboratory data of the 227 patients and the number of patients with each different stage of liver fibrosis are presented in Table 1. In the training group, stages of liver fibrosis using the modified Scheuer scoring system as the reference method were as follows: S0–S1 in 61 individuals (41.50%), S2 in 55 (37.41%), S3 in 25 (17.01%), and S4 in 6 (4.08%). In the validation group, S0–S1 contained 32 individuals (40.00%), S2 in 30 (37.50%), S3 in 14 (17.50%), and S4 in 4 (5.00%). The concentration of LECT2, APRI, and FIB-4 were 4.97, 0.24, and 0.91 in the training group, and 4.76, 0.28, and 0.89 in the validation group, respectively. The main characteristics and the numbers in various stages of liver fibrosis showed no significant differences between the training group and the validation group.

**TABLE 1:** Demographic and laboratory data of patients with CHB in the training group and validation group.

Characteristic	Training group (*n* = 147)	Validation group (*n* = 80)	*p* value
Gender (M/F)	90/57	48/32	0.857
Age	38.00 (32.00, 48.00)	35.50 (31.00, 43.75)	0.080
He-positive, *n*	107	58	1.000
ALT	30.00 (20.00, 50.00)	32.00 (21.25, 64.75)	0.269
AST	23.00 (19.00, 32.00)	25.50 (19.00, 40.75)	0.243
GGT	18.00 (12.00, 30.25)	16.00 (12.00, 25.25)	0.296
Tbil	13.70 (10.80, 17.30)	14.45 (10.38, 17.73)	0.833
ALB	47.60 (45.20, 49.20)	47.10 (44.43, 50.30)	0.779
PLT	196.00 (154.00, 235.00)	203.00 (157.50, 235.00)	0.998
LECT2	4.97 (2.41, 7.65)	4.76 (2.84, 7.26)	0.995
APRI	0.24 (0.18, 0.39)	0.28 (0.21, 0.42)	0.105
FIB-4	0.91 (0.63, 1.34)	0.89 (0.64, 1.30)	0.943
Liver fibrosis, n			0.982^a^
S1	61	32	
S2	55	30	
S3	25	14	
S4	6	4	

Data are expressed as the median and quartiles.

a Fisher’s exact test.

ALT, aspartate aminotransferase (IU/L); AST, alanine aminotransferase (IU/L); PLT, platelet count (×10^9/L); GGT, gamma-glutamyl transpeptidase (U/L); Tbil, total bilirubin (µmol/L); ALB, albumin (g/L); APRI, aspartate aminotransferase-to-platelet ratio index; FIB-4, fibrosis index based on the four factors; LECT2, leukocyte cell-derived chemotaxin 2 (ng/ml).

### Serum LECT2 is Positively Associated With the Stage of Liver Fibrosis

**FIGURE 1:**
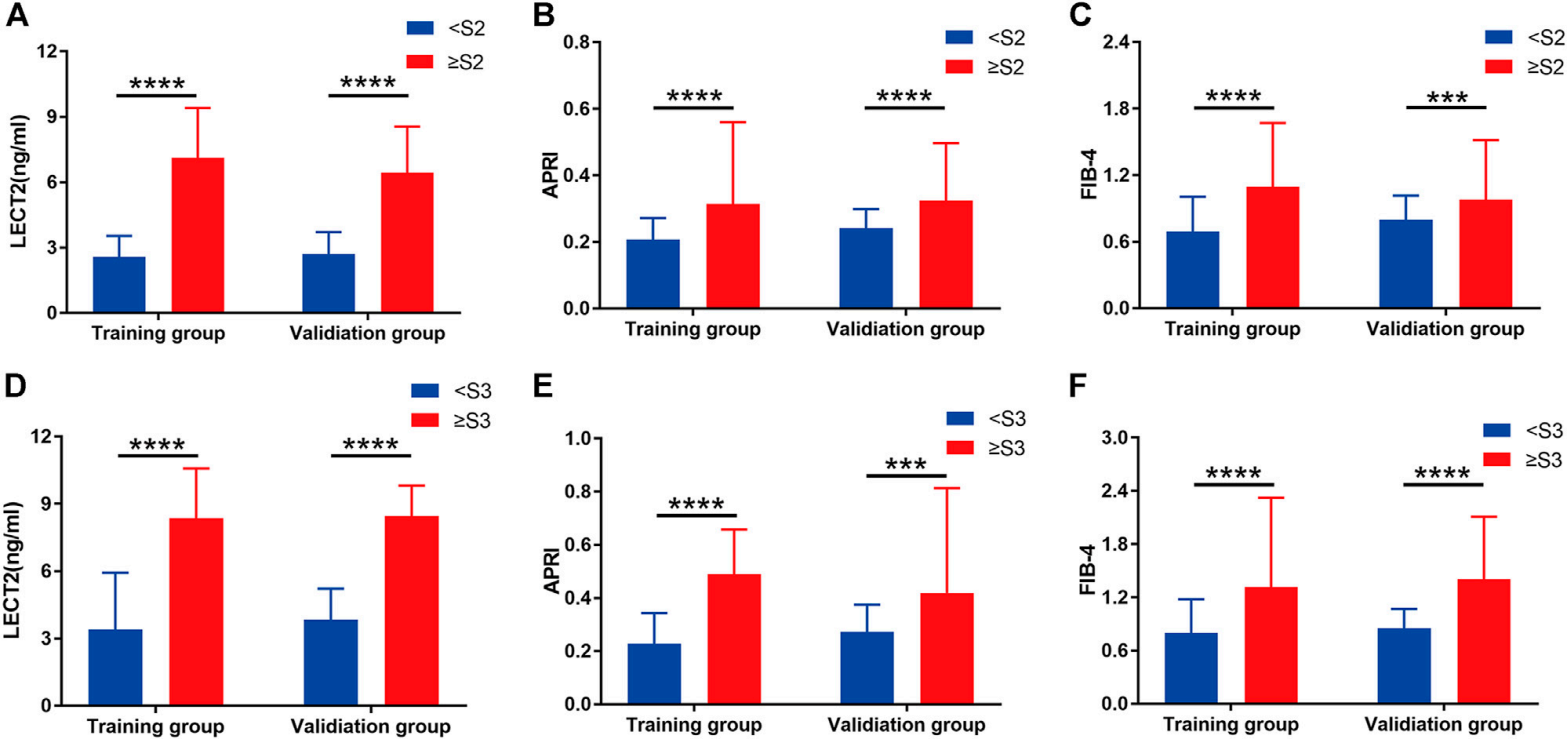
Grouped bar chart depicting the serum liver fibrosis marker levels in patients with CHB in the training group and validation group. **(A)**, **(B)**, and **(C)** show the serum LECT2, APRI, and FIB-4 in the liver fibrosis stage <S2 and ≥S2, respectively. **(D)**, **(E)**, and **(F)** show the serum LECT2, APRI and FIB-4 in the liver fibrosis stage <S3 and ≥S3, respectively. The differences between <S2 and ≥S2 or <S3 and ≥S3 for each of the three liver fibrosis markers were significant. ***p < 0.001, ****p < 0.0001.

The expression of LECT2, APRI, and FIB-4 in various stages of liver fibrosis were shown in Table 2. It is obvious that whether in the training group or the validation group, the level of serum LECT2, APRI, and FIB-4 increased with the aggravation of liver fibrosis (all *p* < 0.05), and the patients with S4 showed the highest LECT2. In the training group, the Spearman correlation coefficient was used to demonstrate that LECT2, APRI, and FIB-4 were positively associated with the degree of liver fibrosis. And the LECT2 was shown to be more positively correlated with the stages of liver fibrosis than the others (*r* = 0.673, 0.423, 0.450 for LECT2, APRI, FIB-4, respectively). Furthermore, we tried to investigate the potential predictors of liver fibrosis through the univariate and multivariate regression analyses. Data showed that LECT2 was the independent predictor of significant liver fibrosis (OR = 2.311, *P* = 0.000) and advanced liver fibrosis (OR = 1.555, *P* = 0.000) (Supplementary Tables S1, S2). In the detailed analysis, we compared the expression of LECT2, APRI, and FIB-4 in the condition of ≥S2 or ≥ S3, respectively. The results showed that the level of these predictors was increased in patients with more serious liver fibrosis in the training group and validation group (Figure 1).

**TABLE 2:** Levels of APRI, FIB-4 and LECT2 in different stages of liver fibrosis in the training group and validation group.

Group		Stages of liver fibrosis				*p* value
		S0-S1	S2	S3	S4	
Training group	APRI	0.21 (0.16, 0.27)	0.25 (0.18, 0.39)	0.43 (0.23, 0.62)	0.65 (0.53, 4.48)	0.000
	FIB-4	0.69 (0.57, 1.00)	1.01 (0.63, 1.52)	1.25 (0.82, 2.24)	1.69 (0.94, 11.65)	0.000
	LECT2	2.57 (1.99, 3.54)	5.93 (3.48, 7.86)	7.74 (6.76, 10.50)	10.48 (8.76, 11.38)	0.000
Validation group	APRI	0.24 (0.16, 0.29)	0.31 (0.24, 0.46)	0.36 (0.28, 0.60)	0.73 (0.33, 1.19)	0.000
	FIB-4	0.79 (0.48, 1.02)	0.88 (0.68, 1.24)	1.38 (0.78, 1.53)	2.65 (1.57, 3.35)	0.000
	LECT2	2.70 (1.68, 3.72)	5.06 (3.99, 6.89)	8.41 (6.31, 9.41)	9.44 (6.86, 14.14)	0.000

Data are expressed as the median and quartiles, APRI: aspartate aminotransferase-to-platelet ratio index; FIB-4: fibrosis index based on the four factors; LECT2: leukocyte cell-derived chemotaxin 2 (ng/ml).

### Diagnostic Performance of LECT2 in all CHB Patients

**FIGURE 2:**
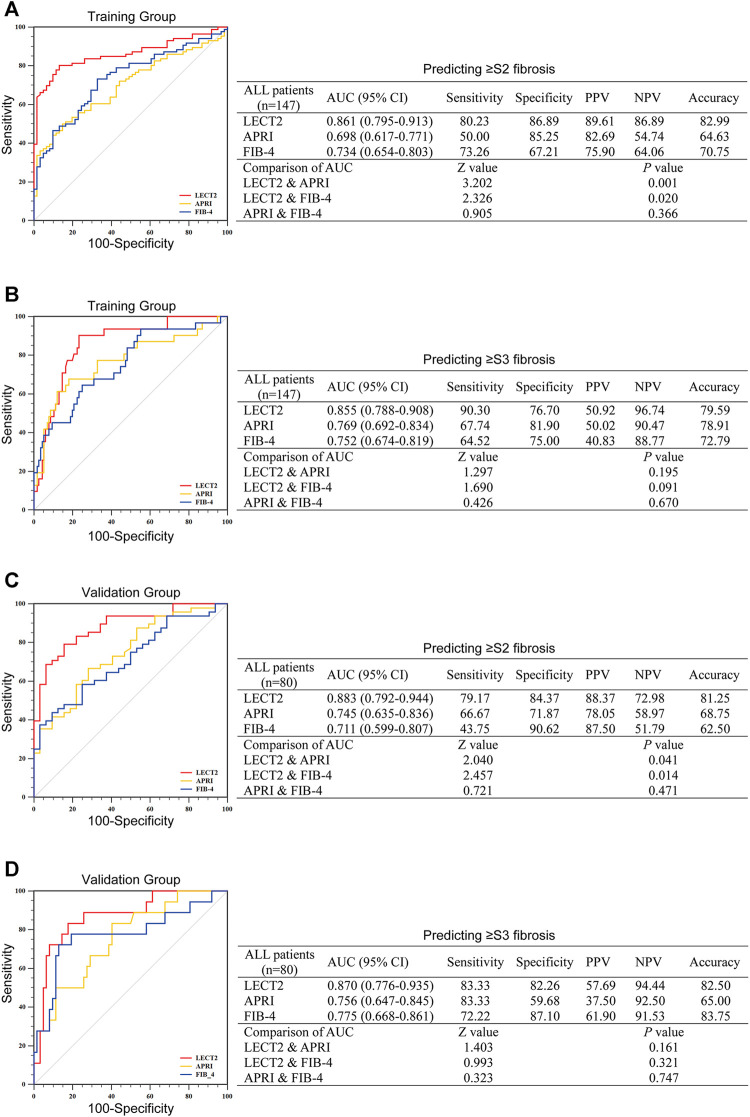
The diagnostic efficacy of LECT2, APRI, and FIB-4 for identifying liver fibrosis in all CHB patients. **(A,B)** Identification of significant liver fibrosis (≥S2) or advanced liver fibrosis (≥S3) in the training group. **(C)** and **(D)** show the further verification of the validation group to the training group. AUC, the area under the ROC; PPV, positive predictive value; NPV, negative predictive value.

As mentioned above, the serum level of LECT2 was upregulated in various liver fibrosis groups, suggesting the possibility that LECT2 could discriminate the liver fibrosis stage. The efficacy of LECT2, APRI, and FIB-4 was compared by calculating the AUC of these predictors. In the training group, for predicting significant liver fibrosis (≥S2), the AUC was 0.861 of LECT2, which was shown to be significantly higher than that of APRI (0.698), and FIB-4 (0.734) (all *p* < 0.05) (Figure 2A). In the validation group, the AUC of LECT2 was also superior to that of APRI, and FIB-4 (0.883, 0.745, 0.711 for LECT2, APRI, FIB-4) (Figure 2C). The optimal cutoff value of LECT2 to predict ≥S2 liver fibrosis was 4.13 and 4.20 ng/ml in the training group and validation group. The diagnostic accuracy of LECT2 in predicting significant liver fibrosis was 82.99 and 81.25% in the training group and validation group, respectively.

We further validated the efficacy of these indicators for the prediction of advanced liver fibrosis (≥S3). In the training group, the AUC of LECT2 was 0.855, which was higher than those of APRI, and FIB-4 (0.769 and 0.752, respectively) (Figure 2B). In the validation group, the AUC was 0.870, 0.756, 0.775 for LECT2, APRI, FIB-4, respectively (Figure 2D). The optimal cutoff value of LECT2 for predicting ≥S3 liver fibrosis was 6.10 ng/ml in the training group and 5.92 ng/ml in the validation group. The accuracy of LECT2 in predicting advanced liver fibrosis was 79.59 and 82.50% in the training and validation group, respectively.

### Diagnostic Performance of LECT2 in CHB Patients With HBeAg-Negative

**FIGURE 3:**
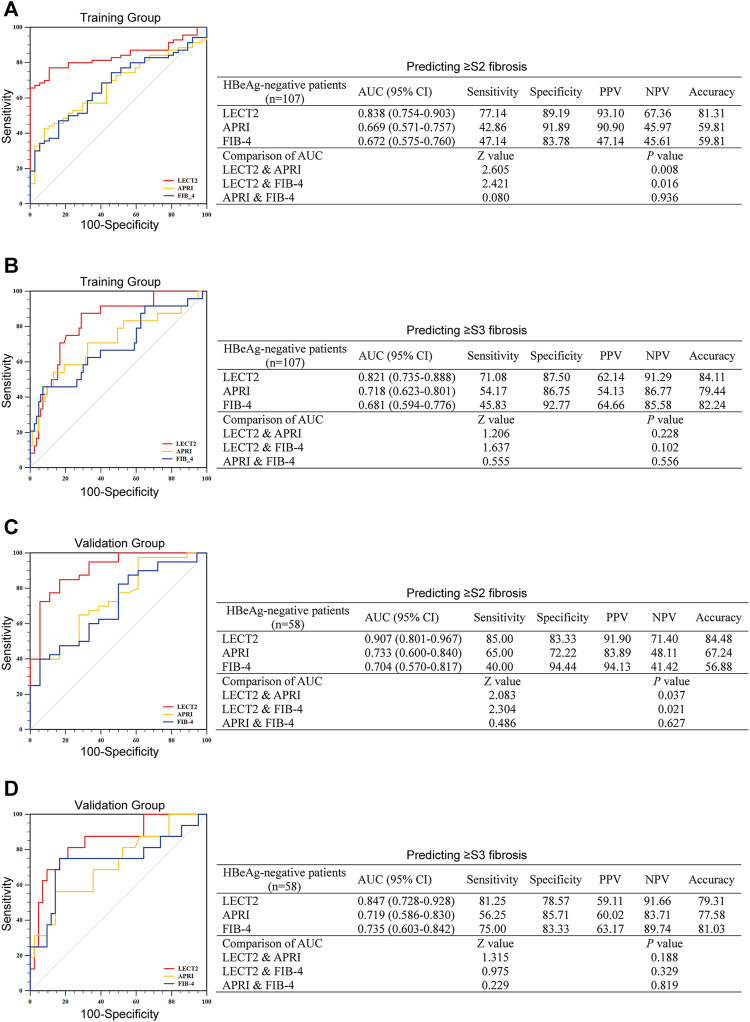
The diagnostic efficacy of LECT2, APRI, and FIB-4 for identifying liver fibrosis in CHB patients with HBeAg-negative. **(A,B)** Identification of significant liver fibrosis (≥S2) or advanced liver fibrosis (≥S3) in the training group. **(C)** and **(D)** show the further verification of the validation group to the training group. AUC, the area under the ROC; PPV, positive predictive value; NPV, negative predictive value.

In the training group, there were 107 CHB patients with HBeAg-negative, and in the validation group, there were 58 CHB patients with HBeAg-negative, respectively. We compared the efficacy of LECT2 in predicting significant liver fibrosis (≥S2) to APRI, FIB-4 in the training group first. As shown in Figure 3A, the AUC of LECT2 (0.838) was higher than that of APRI (0.669) and FIB-4 (0.672) in CHB patients with HBeAg-negative (all *p* < 0.05). Then, we validated the efficacy of LECT2 in the validation group, as shown in Figure 3C, the excellent efficacy of LECT2 had also been demonstrated in the validation group, the AUC was 0.907, 0.733, and 0.704 for LECT2, APRI, and FIB-4 (all *p* < 0.05). The optimal cutoff value of LECT2 to predict ≥ S2 liver fibrosis was 4.11 ng/ml in the training group and 4.20 ng/ml in the validation group. We calculated the accuracy of LECT2 in predicting significant liver fibrosis separately, and it was 81.31 and 84.48% in these two groups, respectively.

For predicting advanced liver fibrosis (≥S3), in the training group, LECT2, APRT, and FIB-4 gave an AUC of 0.821, 0.718, and 0.681, respectively (Figure 3B). The AUC of these predictors in the validation group was the same as the training group (0.847 for LECT2, 0.719 for APRI, and 0.735 for FIB-4) (Figure 3D). In the training group, the optimal cutoff of LECT2 was 6.09 ng/ml and yielded 84.11% accuracy. In the validation group, the optimal cutoff of LECT2 was 5.92 ng/ml, yielding 79.31% accuracy.

### Correlation Between the Protein Levels of LECT2 in the Liver Tissues and Liver Fibrosis Stage

**FIGURE 4:**
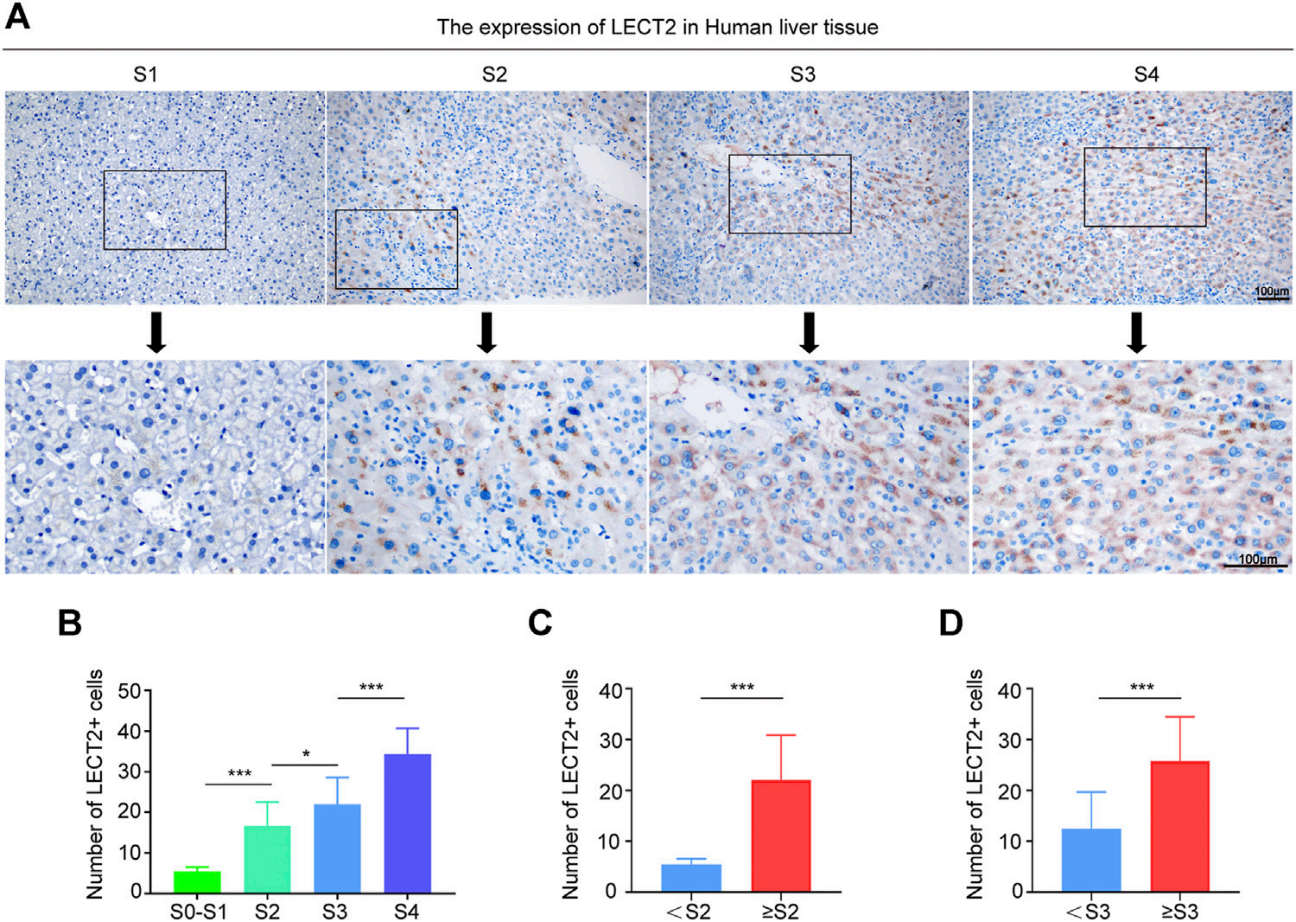
Expression of LECT2 protein levels in the human liver tissue. **(A)** Immunohistochemical staining of LECT2 in liver tissues sections from CHB patients in different stages of liver fibrosis. **(B)**, **(C)**, and **(D)** show the quantitative analysis of LECT2+ cells in **(A)**. *p < 0.05, ***p < 0.001.

To verify that LECT2 is a direct predictor of liver fibrosis, we not only detected serum LECT2 but also examined the protein levels of LECT2 in the liver tissues. The expression of LECT2 was detected from liver tissues through an IHC assay. As shown in Figure 4A, in liver samples with a lower fibrosis stage, lower protein levels of LECT2 were observed in hepatocytes. In contrast, significantly stronger LECT2 expression was observed in the liver tissues with significant (≥S2) and advanced (≥S3) liver fibrosis. The number of LECT2+ cells in different liver fibrosis stages was calculated. It is obvious that with the aggravation of liver fibrosis, the number of LECT2+ cells increased gradually (Figures 4B–D).

### Correlation Between the Levels of LECT2 mRNA in the Liver Tissues and Liver Fibrosis Stage

**FIGURE 5:**
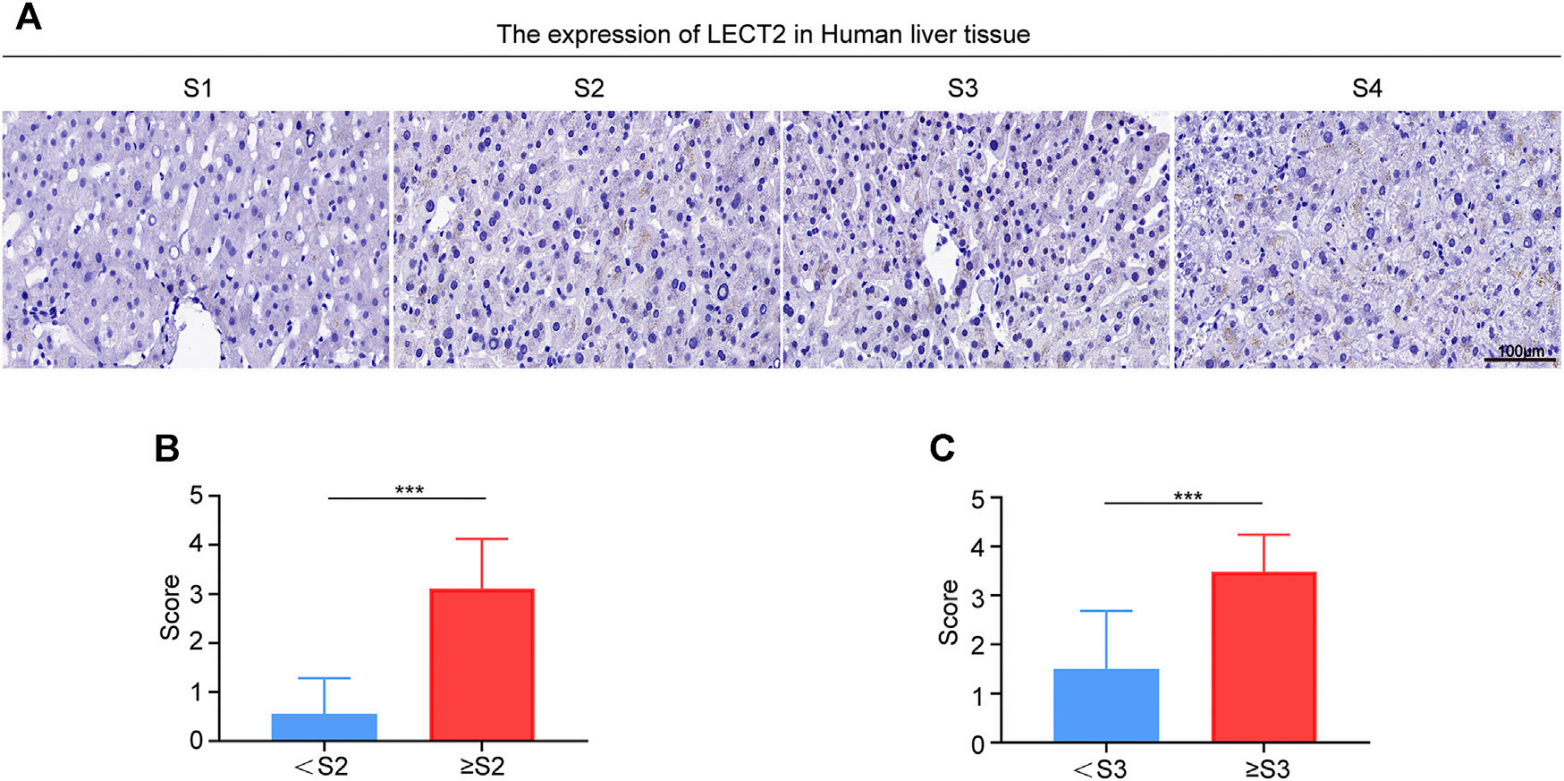
Expression of LECT2 mRNA in the human liver tissue. **(A)** Representative photomicrographs of RNAscope *in situ* hybridization (ISH) showing LECT2 expression in liver tissues sections from CHB patients in different stages of liver fibrosis. **(B)** and **(C)** show the quantitative analysis of the results of RNAscope *in situ* hybridization (ISH) in **(A)**. ***p < 0.001.

In subsequent analysis, we tested the level of LECT2 mRNA in the liver tissues. The staining results of LECT2 mRNA levels revealed that there was a higher abundance of LECT2 mRNA in the advanced fibrosis stage (Figure 5A). We interpreted the results according to the official scoring criteria. The expression of LECT2 mRNA was compared between the <S2 group and the ≥S2 group, data showed that it was significantly higher in the ≥S2 (significant liver fibrosis) group (*p* < 0.01) (Figure 5B). The concentration of LECT2 mRNA in the ≥S3 (advanced liver fibrosis) group was more (*p* < 0.01) than that in the <S2 group (Figure 5C).

## Discussion

No study has investigated the possible relationship between LECT2 and the degree of liver fibrosis in patients with CHB until recently. This is the first report that aimed to figure out the association between the expression of LECT2 and the severity of liver fibrosis. Epidemiological studies have reported that the staging of liver fibrosis is positively linked with significant clinical complications, and an increased liver fibrosis stage is related to higher mortality than in patients without liver fibrosis (Berumen et al., 2021). Besides, the development of histopathology often precedes any overt clinical features and it is usually difficult to find in time in the clinic. The guidelines of CHB also require that treatment should be started once significant liver fibrosis is reached. For CHB patients, it is necessary that they are warned of the severity of liver fibrosis as soon as possible (EASL, 2017).

Liver biopsy is the most classic method to estimate liver fibrosis through expert histological interpretation. But it had some limitations in clinical application due to invasiveness, high price, and multiple complications. Considering the shortcomings of liver biopsy, finding some noninvasive methods to describe the degree of liver fibrosis is a very attractive option. Several noninvasive tests had been reported to predict liver fibrosis in NAFLD and HCV, and so on (Chinnaratha et al., 2014; Boursier et al., 2016). These noninvasive tests include 1) simple blood tests using common parameters, but their accuracy is low; 2) specialized blood tests that are more direct but not widely available and require specialist laboratory assessment; 3) some require costly elastography (Parola and Pinzani, 2019). Hence, there is a need for a new and direct biomarker that is suitable and economical.

Noninvasive scoring systems such as APRI and FIB-4 were used to evaluate the degree of liver fibrosis that had been reported earlier (WHO, 2015). The efficacy of LETC2 in predicting liver fibrosis was checked by comparing it with the above predictors. The results of this study displayed that the AUC of APRI in predicting significant and advanced liver fibrosis was 0.698 and 0.745 in the training group and validation group, respectively. In the study of Lu et al., the AUC of APRI for predicting significant and cirrhosis was 0.66 and 0.72 (Lu et al., 2020), which is consistent with this research. In this study, the ACU of FIB-4 was 0.734 for predicting significant liver fibrosis. Reviewing previous studies, the AUC of FIB-4 for significant liver fibrosis ranged from 0.72 to 0.76 (Xiao et al., 2015; Park et al., 2016; Tseng et al., 2018), and our results fit this range. Besides, several previous articles had reported that the AUC of APRI and FIB-4 for predicting advanced liver fibrosis (≥S3) were 0.717 and 0.760, respectively (Li et al., 2018; Gao et al., 2020). In our study, the ACU of APRI and FIB-4 was 0.769 and 0.752, which seems slightly higher than previous studies. All the above evidence reminds us that APRI and FIB-4 only showed modest predictive performance for the identification of liver fibrosis in CHB patients.

Recently, accumulating evidence has indicated that LECT2 played crucial roles in various diseases, including diabetes (Lan et al., 2014), lung cancer (Hung et al., 2018), liver cancer (Ong et al., 2011; Chen et al., 2014; L’Hermitte et al., 2019), NAFLD (Yoo et al., 2017), and so on. More importantly, our previous studies confirmed for the first time that LECT2 can bind to Tie1 as a ligand to promote the progress of liver fibrosis by affecting angiogenesis, and verified the conclusion *in vitro* and experimental animal models. In the process of studying the mechanism of LECT2 promoting liver fibrosis, we found that serum LECT2 was raised significantly in patients with liver cirrhosis and was increased as the Child-Pugh score progressed from A to D (Xu et al., 2019). However, whether LECT2 can be used as a direct biomarker to diagnose the early stage of liver fibrosis in CHB patients remains unknown.

To verify the efficacy of LECT2, firstly we compared the AUC of LECT2, APRI, FIB-4 in detecting significant (≥S2), and advanced (≥S3) liver fibrosis in CHB patients in the training group. The results showed that the AUC of LECT2 in predicting significant and advanced liver fibrosis was 0.861 and 0.855, which was higher than APRI and FIB-4. The optimal cutoff value of LECT2 is 4.13 ng/ml to predict significant liver fibrosis (≥S2), 6.10 ng/ml to predict advanced liver fibrosis (≥S3). Then, according to the guidelines of CHB (EASL, 2017; Terrault et al., 2018) which have emphasized that the degree of liver fibrosis and the various situations of CHB patients should be taken into full consideration. We discussed the efficacy of LECT2 on the premise of considering different situations of CHB patients, such as HBeAg-negative and ALT (ALT < ULN or ALT < 2ULN). We calculated and compared the diagnostic efficacy of LECT2, APRI, and FIB-4 in patients with HBeAg-negative, ALT < ULN or ALT < 2ULN (data shown in the Supplementary Figure S1), respectively. Beyond these different grouping conditions, the efficacy of LECT2 to predict significant and advanced liver fibrosis was still superior to APRI and FIB-4. Furthermore, we verified the efficacy of LECT2 in the validation group which was consistent with the training group, the AUC of LECT2 was 0.883 and 0.870 for significant and advanced liver fibrosis, respectively. All these results indicated that LECT2 is good at detecting liver fibrosis in CHB patients.

Our data showed that there was an obvious correlation between the high expression of serum LECT2 and the deterioration of liver fibrosis. Moreover, we detected the expression of protein and mRNA levels of LECT2 in CHB patient liver samples using IHC analysis. Our results showed that the LECT2 protein levels and mRNA levels of LECT2 in hepatic tissue were upregulated in CHB patients who were diagnosed with significant and advanced liver fibrosis by liver biopsy. It seems that LECT2 is more reliable than APRI and FIB-4 since these were obtained by calculating some liver-related laboratory indicators. More importantly, LECT2 is a protein secreted by hepatocytes and expressed in both serum and tissues. It means that LECT2 could be easily detected from serum by ELISA or flow cytometry and did not require additional histological interpretation, which is a huge advantage of LECT2 in clinical application.

In conclusion, LECT2 is a novel and direct predictor, which is suitable as a screening biomarker for significant and advanced liver fibrosis, and the diagnostic efficacy of LECT2 in different situations of patients with CHB had been confirmed. The detection of LECT2 contributes to the more accurate evaluation of liver fibrosis.

## Data Availability

The original contributions presented in the study are included in the article/Supplementary Material, further inquiries can be directed to the corresponding authors.

## References

[B1] Bertrais S. Boursier J. Ducancelle A. Oberti F. Fouchard‐Hubert I. Moal V. (2017). Prognostic Durability of Liver Fibrosis Tests and Improvement in Predictive Performance for Mortality by Combining Tests. J. Gastroenterol. Hepatol. 32 (6), 1240–1249. 10.1111/jgh.13668 27897323

[B2] Berumen J. Baglieri J. Kisseleva T. Mekeel K. (2021). Liver Fibrosis: Pathophysiology and Clinical Implications. WIREs Mech. Dis. 13 (1), e1499. 10.1002/wsbm.1499 PMC9479486 32713091

[B3] Boursier J. Vergniol J. Guillet A. Hiriart J.-B. Lannes A. Le Bail B. (2016). Diagnostic Accuracy and Prognostic Significance of Blood Fibrosis Tests and Liver Stiffness Measurement by FibroScan in Non-alcoholic Fatty Liver Disease. J. Hepatol. 65 (3), 570–578. 10.1016/j.jhep.2016.04.023 27151181

[B4] Cao Q. Lu X. Azad B. B. Pomper M. Smith M. He J. (2020). cis-4-[18F]fluoro-L-proline Molecular Imaging Experimental Liver Fibrosis. Front. Mol. Biosci. 7, 90. 10.3389/fmolb.2020.00090 32500081 PMC7243806

[B5] Chen C.-K. Yang C.-Y. Hua K.-T. Ho M.-C. Johansson G. Jeng Y.-M. (2014). Leukocyte Cell-Derived Chemotaxin 2 Antagonizes MET Receptor Activation to Suppress Hepatocellular Carcinoma Vascular Invasion by Protein Tyrosine Phosphatase 1B Recruitment. Hepatology 59 (3), 974–985. 10.1002/hep.26738 24114941

[B6] Chinnaratha M. A. Jeffrey G. P. MacQuillan G. Rossi E. de Boer B. W. Speers D. J. (2014). Prediction of Morbidity and Mortality in Patients with Chronic Hepatitis C by Non-invasive Liver Fibrosis Models. Liver Int. 34 (5), 720–727. 10.1111/liv.12306 24034439

[B7] Cooke G. S. Andrieux-Meyer I. Applegate T. L. Atun R. Burry J. R. Cheinquer H. (2019). Accelerating the Elimination of Viral Hepatitis: a Lancet Gastroenterology & Hepatology Commission. Lancet Gastroenterol. Hepatol. 4 (2), 135–184. 10.1016/S2468-1253(18)30270-X 30647010

[B8] Demler O. V. Pencina M. J. D'Agostino R. B. (2012). Misuse of DeLong Test to Compare AUCs for Nested Models. Statist. Med. 31 (23), 2577–2587. 10.1002/sim.5328 PMC3684152 22415937

[B9] Dulai P. S. Singh S. Patel J. Soni M. Prokop L. J. Younossi Z. (2017). Increased Risk of Mortality by Fibrosis Stage in Nonalcoholic Fatty Liver Disease: Systematic Review and Meta-Analysis. Hepatology 65 (5), 1557–1565. 10.1002/hep.29085 28130788 PMC5397356

[B10] European Association for the Study of the Liver (2017). EASL 2017 Clinical Practice Guidelines on the Management of Hepatitis B Virus Infection. J. Hepatol. 67 (2), 370–398. 10.1016/j.jhep.2017.03.021 28427875

[B11] Gao F. Li K. Li Y. Ding G. Q. Lu X. L. Wang H. (2020). Serum miR-17 Levels in Patients with Hepatitis B Virus Induced Liver Fibrosis. Eur. Rev. Med. Pharmacol. Sci. 24 (11), 6245–6251. 10.26355/eurrev_202006_21522 32572891

[B13] Hung W.-Y. Chang J.-H. Cheng Y. Chen C.-K. Chen J.-Q. Hua K.-T. (2018). Leukocyte Cell-Derived Chemotaxin 2 Retards Non-small Cell Lung Cancer Progression through Antagonizing MET and EGFR Activities. Cell Physiol Biochem 51 (1), 337–355. 10.1159/000495233 30453282

[B14] Jia J. Hou J. Ding H. Chen G. Xie Q. Wang Y. (2015). Transient Elastography Compared to Serum Markers to Predict Liver Fibrosis in a Cohort of C Hinese Patients with Chronic Hepatitis B. J. Gastroenterol. Hepatol. 30 (4), 756–762. 10.1111/jgh.12840 25353058

[B15] Jung T. W. Chung Y. H. Kim H.-C. Abd El-Aty A. M. Jeong J. H. (2018). LECT2 Promotes Inflammation and Insulin Resistance in Adipocytes via P38 Pathways. J. Mol. Endocrinol. 61 (1), 37–45. 10.1530/JME-17-0267 29650721

[B16] Lan F. Misu H. Chikamoto K. Takayama H. Kikuchi A. Mohri K. (2014). LECT2 Functions as a Hepatokine that Links Obesity to Skeletal Muscle Insulin Resistance. Diabetes 63 (5), 1649–1664. 10.2337/db13-0728 24478397

[B17] L’Hermitte A. Pham S. Cadoux M. Couchy G. Caruso S. Anson M. (2019). Lect2 Controls Inflammatory Monocytes to Constrain the Growth and Progression of Hepatocellular Carcinoma. Hepatology 69 (1), 160–178. 10.1002/hep.30140 30070727

[B18] Li J. Mao R.-C. Li X.-L. Zheng J.-W. Qi X. Yuan Q. (2018). A Novel Noninvasive index for the Prediction of Moderate to Severe Fibrosis in Chronic Hepatitis B Patients. Dig. Liver Dis. 50 (5), 482–489. 10.1016/j.dld.2017.12.028 29396134

[B19] Loomba R. Adams L. A. (2020). Advances in Non-invasive Assessment of Hepatic Fibrosis. Gut 69 (7), 1343–1352. 10.1136/gutjnl-2018-317593 32066623 PMC7945956

[B20] Lu X.-J. Yang X.-J. Sun J.-Y. Zhang X. Yuan Z.-X. Li X.-H. (2020). FibroBox: a Novel Noninvasive Tool for Predicting Significant Liver Fibrosis and Cirrhosis in HBV Infected Patients. Biomark Res. 8, 48. 10.1186/s40364-020-00215-2 33005419 PMC7520974

[B21] Ma S. Xie Z. Zhang L. Yang Y. Jiang H. Ouyang X. (2021). Identification of a Potential miRNA-mRNA Regulatory Network Associated with the Prognosis of HBV-ACLF. Front. Mol. Biosci. 8, 657631. 10.3389/fmolb.2021.657631 33996909 PMC8113841

[B22] McGill D. B. Rakela J. Zinsmeister A. R. Ott B. J. (1990). A 21-year Experience with Major Hemorrhage after Percutaneous Liver Biopsy. Gastroenterology 99 (5), 1396–1400. 10.1016/0016-5085(90)91167-5 2101588

[B23] Moon A. M. Singal A. G. Tapper E. B. (2020). Contemporary Epidemiology of Chronic Liver Disease and Cirrhosis. Clin. Gastroenterol. Hepatol. 18 (12), 2650–2666. 10.1016/j.cgh.2019.07.060 31401364 PMC7007353

[B24] Okumura A. Saito T. Tobiume M. Hashimoto Y. Sato Y. Umeyama T. (2017). Alleviation of Lipopolysaccharide/D -Galactosamine-Induced Liver Injury in Leukocyte Cell-Derived Chemotaxin 2 Deficient Mice. Biochem. Biophys. Rep. 12, 166–171. 10.1016/j.bbrep.2017.09.011 29090278 PMC5645298

[B25] Ong H. T. Tan P. K. Wang S. M. Hian Low D. T. Pj Ooi L. L. Hui K. M. (2011). The Tumor Suppressor Function of LECT2 in Human Hepatocellular Carcinoma Makes it a Potential Therapeutic Target. Cancer Gene Ther. 18 (6), 399–406. 10.1038/cgt.2011.5 21394108

[B26] Park M. S. Kim S. W. Yoon K. T. Kim S. U. Park S. Y. Tak W. Y. (2016). Factors Influencing the Diagnostic Accuracy of Acoustic Radiation Force Impulse Elastography in Patients with Chronic Hepatitis B. Gut Liver 10 (2), 275–282. 10.5009/gnl14391 26087790 PMC4780458

[B27] Parola M. Pinzani M. (2019). Liver Fibrosis: Pathophysiology, Pathogenetic Targets and Clinical Issues. Mol. aspects Med. 65, 37–55. 10.1016/j.mam.2018.09.002 30213667

[B28] Polaris Observatory Collaborators (2018). Global Prevalence, Treatment, and Prevention of Hepatitis B Virus Infection in 2016: a Modelling Study. Lancet Gastroenterol. Hepatol. 3 (6), 383–403. 10.1016/S2468-1253(18)30056-6 29599078

[B29] Revill P. A. Chisari F. V. Block J. M. Dandri M. Gehring A. J. Guo H. (2019). A Global Scientific Strategy to Cure Hepatitis B. Lancet Gastroenterol. Hepatol. 4 (7), 545–558. 10.1016/S2468-1253(19)30119-0 30981686 PMC6732795

[B30] Segawa Y. Itokazu Y. Inoue N. Saito T. Suzuki K. (2001). Possible Changes in Expression of Chemotaxin LECT2 mRNA in Mouse Liver after Concanavalin A-Induced Hepatic Injury. Biol. Pharm. Bull. 24 (4), 425–428. 10.1248/bpb.24.425 11305608

[B31] Singh S. Muir A. J. Dieterich D. T. Falck-Ytter Y. T. (2017). American Gastroenterological Association Institute Technical Review on the Role of Elastography in Chronic Liver Diseases. Gastroenterology 152 (6), 1544–1577. 10.1053/j.gastro.2017.03.016 28442120

[B32] Slowik V. Borude P. Borude P. Jaeschke H. Woolbright B. L. Lee W. M. (2019). Leukocyte Cell Derived Chemotaxin-2 (Lect2) as a Predictor of Survival in Adult Acute Liver Failure. Transl. Gastroenterol. Hepatol. 4, 17. 10.21037/tgh.2019.03.03 30976720 PMC6458334

[B33] Sterling R. K. Lissen E. Clumeck N. Sola R. Correa M. C. Montaner J. (2006). Development of a Simple Noninvasive index to Predict Significant Fibrosis in Patients with HIV/HCV Coinfection. Hepatology 43 (6), 1317–1325. 10.1002/hep.21178 16729309

[B34] Takata N. Ishii K.-A. Takayama H. Nagashimada M. Kamoshita K. Tanaka T. (2021). LECT2 as a Hepatokine Links Liver Steatosis to Inflammation via Activating Tissue Macrophages in NASH. Sci. Rep. 11 (1), 555. 10.1038/s41598-020-80689-0 33436955 PMC7804418

[B35] Terrault N. A. Lok A. S. F. McMahon B. J. Chang K.-M. Hwang J. P. Jonas M. M. (2018). Update on Prevention, Diagnosis, and Treatment of Chronic Hepatitis B: AASLD 2018 Hepatitis B Guidance. Hepatology 67 (4), 1560–1599. 10.1002/hep.29800 29405329 PMC5975958

[B36] Tseng C.-H. Chang C.-Y. Mo L.-R. Lin J.-T. Tai C.-M. Perng D.-S. (2018). Acoustic Radiation Force Impulse Elastography with APRI and FIB-4 to Identify Significant Liver Fibrosis in Chronic Hepatitis B Patients. Ann. Hepatol. 17 (5), 789–794. 10.5604/01.3001.0012.3137 30145564

[B37] Vallet-Pichard A. Mallet V. Nalpas B. Verkarre V. Nalpas A. Dhalluin-Venier V. (2007). FIB-4: an Inexpensive and Accurate Marker of Fibrosis in HCV Infection. Comparison with Liver Biopsy and Fibrotest. Hepatology 46 (1), 32–36. 10.1002/hep.21669 17567829

[B38] Wai C. Greenson J. K. Fontana R. J. Kalbfleisch J. D. Marrero J. A. Conjeevaram H. S. (2003). A Simple Noninvasive index Can Predict Both Significant Fibrosis and Cirrhosis in Patients with Chronic Hepatitis C. Hepatology 38 (2), 518–526. 10.1053/jhep.2003.50346 12883497

[B12] Who (2015). Guidelines for the prevention, care and treatment of persons with chronic hepatitis B infection. Available at: https://apps.who.int/iris/handle/10665/154590 . 26225396

[B39] Who (2016). Combating Hepatitis B and C to Reach Elimination by 2030. Geneva: World Health Organization. Available at: https://www.who.int/publications/i/item/combating-hepatitis-b-and-c-to-reach-elimination-by-2030external icon .

[B40] Who (2017). Global Hepatitis Report, 2017. Geneva, Switzerland: World Health Organization. Available at: http://apps.who.int/iris/bitstream/10665/255016/1/9789241565455-eng.pdf (accessed May 3, 2017).

[B41] Who (2021). World Health Organization. Global Progress Report on HIV, Viral Hepatitis and Sexually Transmitted Infections. Available at: https://www.who.int/publications/i/item/9789240027077external icon .

[B42] Xiao G. Yang J. Yan L. (2015). Comparison of Diagnostic Accuracy of Aspartate Aminotransferase to Platelet Ratio index and Fibrosis-4 index for Detecting Liver Fibrosis in Adult Patients with Chronic Hepatitis B Virus Infection: a Systemic Review and Meta-Analysis. Hepatology 61 (1), 292–302. 10.1002/hep.27382 25132233

[B43] Xu M. Xu H.-H. Lin Y. Sun X. Wang L.-J. Fang Z.-P. (2019). LECT2, a Ligand for Tie1, Plays a Crucial Role in Liver Fibrogenesis. Cell 178 (6), 1478–1492. 10.1016/j.cell.2019.07.021 31474362

[B44] Yan Y. Xing X. Lu Q. Wang X. Luo X. Yang L. (2020). Assessment of Biopsy Proven Liver Fibrosis by Two-Dimensional Shear Wave Elastography in Patients with Primary Biliary Cholangitis. Dig. Liver Dis. 52 (5), 555–560. 10.1016/j.dld.2020.02.002 32111390

[B45] Yoo H. J. Hwang S. Y. Choi J.-H. Lee H. J. Chung H. S. Seo J.-A. (2017). Association of Leukocyte Cell-Derived Chemotaxin 2 (LECT2) with NAFLD, Metabolic Syndrome, and Atherosclerosis. PloS one 12 (4), e0174717. 10.1371/journal.pone.01747110.1371/journal.pone.0174717 28376109 PMC5380318

