# Supplementary figures and images for: LECT2, A Novel and Direct Biomarker of Liver Fibrosis in Patients With CHB
Source: Front Mol Biosci. 2021 Sep 22;8:749648. doi: 10.3389/fmolb.2021.749648 (PMC8492992; doi:10.3389/fmolb.2021.749648)

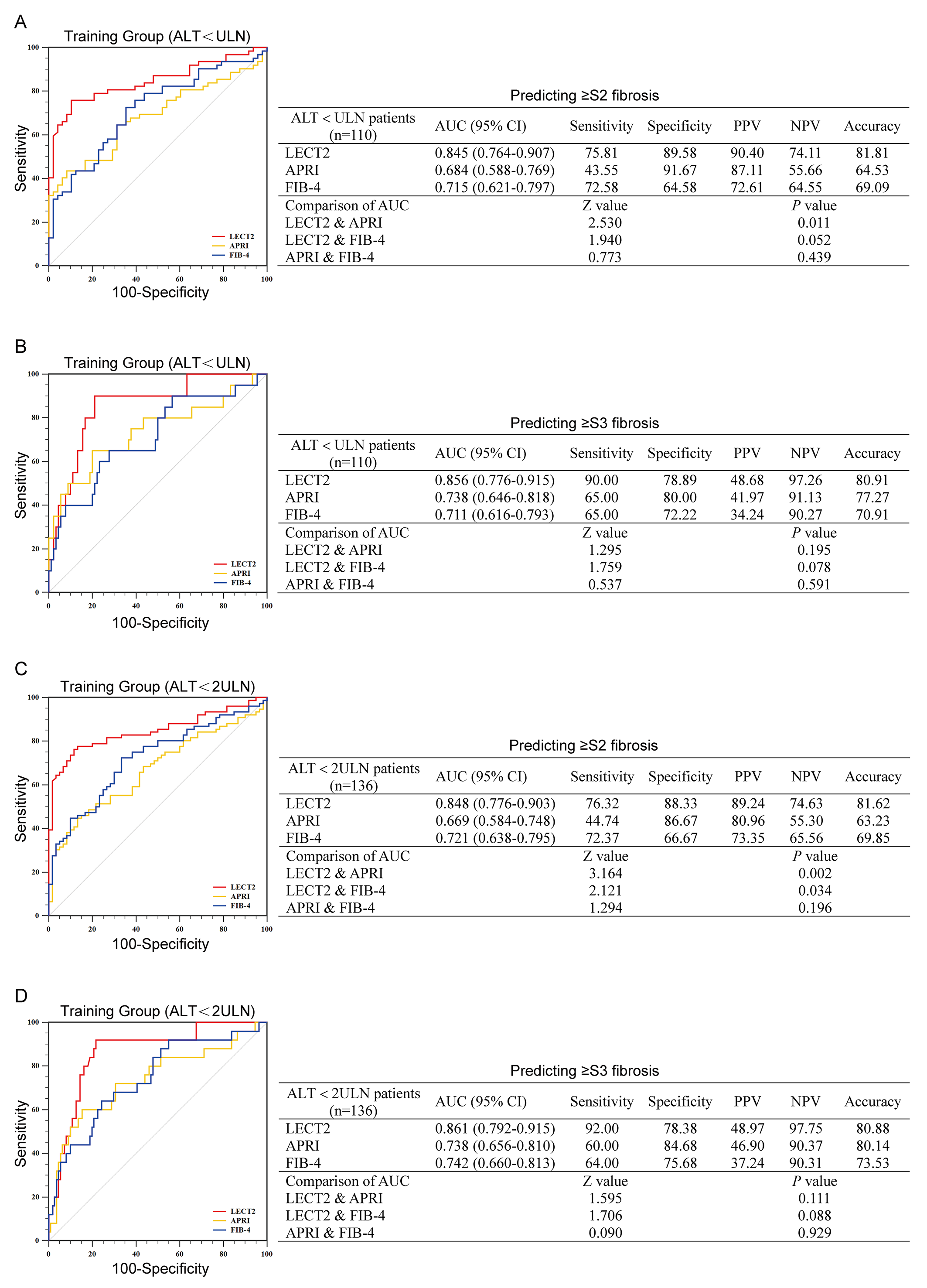

Supplement: Supplementary file 1 [file Image1.TIF]
