# Supplementary material for: LECT2, A Novel and Direct Biomarker of Liver Fibrosis in Patients With CHB
Source: Front Mol Biosci. 2021 Sep 22;8:749648. doi: 10.3389/fmolb.2021.749648 (PMC8492992; doi:10.3389/fmolb.2021.749648)
Supplement: Supplementary file 2 [file DataSheet1.PDF]

Supplementary table 1. univariate and multivariate analyses of baseline characteristics in the liver fibrosis <S2 and ≥S2 in the training group.

	Univariate			Multivariate		
	OR	95% CI	*P* value	OR	95% CI	*P* value
Age	1.065	1.026 to 1.105	0.001	1.060	0.953 to 1.179	0.280
Gender	0.600	0.306 to 1.176	0.137	1.831	0.551 to 6.084	0.324
ALT	1.014	1.001 to 1.027	0.034	0.997	0.937 to 1.062	0.936
AST	1.037	1.008 to 1.068	0.014	0.950	0.791 to 1.139	0.577
GGT	1.021	1.002 to 1.041	0.028	1.004	0.978 to 1.029	0.785
Tbil	1.013	0.972 to 1.056	0.531	1.004	0.952 to 1.060	0.876
ALB	1.003	0.916 to 1.099	0.943	1.097	0.921 to 1.308	0.298
PLT	0.989	0.983 to 0.995	0.000	1.000	0.977 to 1.024	0.996
LECT2	2.045	1.624 to 2.575	0.000	2.311	1.688 to 3.165	0.000
APRI	1.057	1.027 to 1.087	0.000	1.066	0.897 to 1.267	0.467
FIB-4	1.205	1.101 to 1.318	0.000	1.117	0.739 to 1.688	0.600

Supplementary table 2. univariate and multivariate analyses of baseline characteristics in the liver fibrosis <S3 and ≥S3 in the training group.

	Univariate			Multivariate		
	OR	95% CI	*P* value	OR	95% CI	*P* value
Age	1.057	1.015 to 1.100	0.007	1.032	0.930 to 1.144	0.554
Gender	0.358	0.154 to 0.965	0.042	0.496	0.131 to 1.875	0.302
ALT	1.018	1.006 to 1.030	0.003	0.996	0.945 to 1.051	0.894
AST	1.037	1.012 to 1.063	0.004	1.029	0.919 to 1.151	0.624
GGT	1.021	1.006 to 1.037	0.007	1.012	0.989 to 1.036	0.311
Tbil	0.991	0.942 to 1.043	0.726	0.971	0.886 to 1.065	0.535
ALB	0.908	0.815 to 1.011	0.078	1.043	0.866 to 1.257	0.657
PLT	0.983	0.976 to 0.991	0.000	0.986	0.971 to 1.002	0.077
LECT2	1.582	1.339 to 1.869	0.000	1.555	1.279 to 1.892	0.000
APRI	1.040	1.017 to 1.062	0.000	0.998	0.909 to 1.097	0.973
FIB-4	1.133	1.059 to 1.212	0.000	1.016	0.795 to 1.299	0.899
